# Enhanced patient reported outcome measurement suitable for head and neck cancer follow-up clinics

**DOI:** 10.1186/1758-3284-4-32

**Published:** 2012-06-13

**Authors:** Naseem Ghazali, Derek Lowe, Simon N Rogers

**Affiliations:** 1 Regional Maxillofacial Unit, University Hospital Aintree, Liverpool, L9 1AE, UK; 2 Evidence-Based Practice Research Centre (EPRC), Faculty of Health, Edge Hill University, St Helens Road, Ormskirk, L39 4QP, UK

**Keywords:** Health-related quality of life, Head and neck cancer, UW-QOL, Patients Concerns Inventory, Unmet needs

## Abstract

**Background:**

The ‘Worse-Stable-Better’ (W-S-B) question was introduced to capture patient-perceived change in University of Washington Quality of Life (UW-QOL) domains.

**Methods:**

202 head and neck cancer patients in remission prospectively completed UW-QOL and Patients Concerns Inventory (PCI). For each UW-QOL domain, patients indicated whether over the last month things had worsened (W), remained stable (S) or were better (B).

**Results:**

202 patients at 448 attendances selected 1752 PCI items they wanted to discuss in consultation, and 58% (1024/1752) of these were not covered by the UW-QOL. UW-QOL algorithms highlighted another 440 significant problems that the patient did not want to discuss (i.e. the corresponding items on the PCI were not selected).

After making allowance for UW-QOL algorithms to identify 'significant problems' and PCI selection of corresponding issues for discussion there remained clear residual and notable variation in W-S-B responses, in particular to identify patients with significant problems that were getting worse, and patients without significant problems that wanted to discuss issues that were getting worse. Changes in mean UW-QOL scores were notably lower for those getting worse on the W-S-B question, typically by 10 or more units a magnitude that suggests clinically important changes in score.

**Conclusions:**

The W-S-B question adds little questionnaire burden and could help to better identify patients who might benefit from intervention. The results of this study suggest that the UW-QOL with the W-S-B modification should be used together with the PCI to allow optimal identification of issues for patient-clinician discussion during routine outpatient clinics.

## Introduction

Communication between patient and clinician is critical to patient-centred care in oncology [1]. Nevertheless, there are many barriers to effective communication. Within a head and neck cancer (HNC) setting some patients are unwilling to disclose worries and to discuss sensitive issues [2]. Patients with lower self-esteem can find clinical settings intimidating and are unable to voice their concerns, despite regular attendance to these clinics [3]. Furthermore, clinic settings can be hectic and demanding. Patients may be anxious or unwell and may have to wait a long time before being seen. Clinicians are under pressure to perform cancer surveillance tasks, examine prosthesis/wounds and provide information, advice and reassurances during this small window of opportunity. Thus, some patient issues or concerns may be missed completely [4] or only superficially addressed [5], resulting in unmet needs.

Post-treatment HNC survivors suffer morbidity from functional deficits and facial disfigurement, live with considerable symptom burden and experience substantial psychosocial issues [6-11]. For some, worries about cancer recurrence and uncertainties regarding their future may significantly interfere with adjusting to life beyond cancer [7,12,13]. Thus, HNC is a suitable model for developing tools in shared decision-making during survivorship because the patient-centred supportive care required is often resource intensive and multidisciplinary [14].

Health-related quality of life (HRQOL) is usually seen as a secondary outcome measure for patients receiving HNC treatment at the group level to compliment cure and survival rates [15]. More recently, HRQOL measures have been incorporated into routine clinical oncology practice to monitor individual patients [16], and used to screen individuals for significant problems thereby triggering healthcare intervention [17]. Routine use of HRQOL in oncology follow-up clinic consultations has also aided patient-clinician communication [18-20]. By using HRQOL more concerns can be discussed during patient-clinician consultations [19,20], suggesting that HRQOL tools can facilitate the expression of felt needs by directing discussions toward these expressed needs, thereby improving the efficiency of and satisfaction with the consultation.

Not all HRQOL assessment tools consider the relative importance of domains to the patient and patient perceptions of importance may not be reflected in how time is spent discussing specific concerns during a consultation. Also, HRQOL questionnaires are restricted by their wording and often just focus on dysfunction over a relatively short period of time. The rigidity of some HRQOL assessment tools may not accommodate the coping and adaptation that occurs with time. Parallel cognitive and emotional processing during coping and adaptation [21] can result in incongruence between the degree of dysfunction and the significance patients place upon it [22]. For example, patients with significant dysfunction may continue to have poor HRQOL scores even though they have positively adapted to their condition and consequently may not feel any need to discuss it further. Alternatively, patients with very mild dysfunction may have reasonably good HRQOL scores but yet feel a strong need for supportive care in relation to this. Thus, HRQOL tools alone may miss the absence or presence of a felt need to discuss the issue during consultation.

The Liverpool Patients Concern Inventory (PCI) provides a simple, practical tool by which patients can highlight their concerns and needs for discussion in their consultations [23]. The concept of PCI was developed from recognising that the routine application of HRQOL questionnaires was inadequate as a means of identifying individual patient concerns, needs and priorities in outpatient settings. At Aintree hospital, the PCI has been used alongside the University of Washington Quality of life version 4 (UW-QOL) [24], as a means not only of enabling patients to influence the content of their imminent discussion but also to provide the clinician with a fuller profile of information to help guide the consultation. For a particular patient this information yield could include significant UW-QOL problems the patient wants to discuss, significant UW-QOL problems the patient doesn't want to discuss, less significant issues on the UW-QOL the patient wants to discuss, and other concerns beyond the scope of the UW-QOL that the patient wants to discuss.

The PCI and UW-QOL help to focus consultations to make them cover key issues for patients without undue delay to the consultation. The aim of this study was to introduce into this mix of information a ‘Worse-Stable-Better’ (W-S-B) aspect to the UW-QOL version 4 and to see if this additional information contributed uniquely and usefully to the overall information yield available to the clinician at the outset of consultation.

## Methods

The study population comprised HNC patients of one consultant (SNR) attending outpatient clinics from 1st August 2007 to 8^th^ July 2009. Patients on the Liverpool oncology database were included if disease-free and under routine follow-up at least 6 weeks after completing treatment. Patients were excluded if they were before treatment, palliative, attending for other post-operative wound management or were part of another outcomes study in clinic.

Subjects were recruited prospectively while waiting to be seen in the clinic waiting area. A touch-screen computer package comprised the UW-QOL [25] and the Patient Concerns Inventory (PCI)) [23], with the program written in Microsoft ACCESS. Patients entered their own data via the touch screen and their data then went directly onto the hospital drive and using normal password protection arrangements was retrievable by the consultant in another room in clinic immediately before seeing the patient. Only three patients that were approached refused to take part.

The Liverpool Patients Concerns Inventory (PCI) is a holistic, patient-reported tool consisting of 54 issues (45 items from August 2007; 9 more from April 2008) and a list of 15 professionals (healthcare and non-healthcare). The PCI covers a range of issues including hearing, intimacy, fatigue, financial/benefits, PEG tube, relationships, regret, support for family, wound healing, and spiritual/religious aspects. It is completed before the clinic consultation, enabling patients and their carers to highlight concerns they would like to discuss during the clinic visit, and to indicate which professionals they would like to meet or be referred to. The content of the PCI was formulated through the synthesis of items from general and HNC-specific HRQOL questionnaires and discussions with local and national focus groups of HNC patients and professionals involved in the care of HNC patients, namely, the laryngectomy support group, HN support group, patient research forum, hospital volunteers, ward and outpatient staff and the multidisciplinary HNC team. Content validity was evaluated by the Liverpool HNC support group and as a result of this exercise, the extra 9 items were added in April 2008.

The UW-QOL questionnaire [24] consists of 12 single question domains, these having between 3 and 6 response options that are scaled evenly from 0 (worst) to 100 (best) according to the hierarchy of response. Another question asks patients to choose up to three of these domains that have been the most important to them in the previous week. Patients were also asked to state for each UW-QOL domain whether things had got worse (W), stayed the same (S) or got better (B) over the last month. August 2007 was the first time this W-S-B question had been asked of patients and the exact wording was ‘*For each domain, please indicate if things had got better, stayed the same or had got worse in the last month*’.

The analyses for this paper were three-fold. First, W-S-B responses for each domain in relation to clinical and patient characteristics were tested using the Chi-squared test. Second, W-S-B responses were stratified by whether patients had significant problems on the UW-QOL and by whether corresponding PCI items were selected, and association between ordinal W-S-B responses and the 4 subgroups was tested using the Kruskal-Wallis test. We defined a ‘significant problem’ on each UW-QOL domain using information from domain scores and domain importance and by adopting algorithms (see footnote to Table 1) derived in earlier work [17]. Third, UW-QOL domain scores of pairs of clinic visits up to 6 months apart were stratified by W-S-B response at the later clinic - these data were not independent as some patients had more pairs of clinics than others and hence no statistical tests were performed. Clinical-demographic data came from the Liverpool Head and Neck Cancer database. Many statistical tests were performed during the analyses and because of this we have regarded statistical significance as P < 0.01. As the UW-QOL questionnaire is integrated into routine clinical practice in this setting, this study was approved by the University Hospital Aintree Clinical Audit department in the context of service evaluation.

**Table 1:** UW-QOL W-S-B results, in relation to UW-QOL defined significant problems and corresponding items selected on the PCI for discussion at the consultation

		With ‘significant’ UW-QOL problem*		With No ‘significant’ UW-QOL problem*		**Kruskal-Wallis test P value**
		**Corresponding items raised on PCI for discussion in consultation**		**Corresponding items raised on PCI for discussion in consultation**		
		**Yes**	**No**	**Yes**	**No**	
Pain	Worse	18	7	5	5	
	Stable	9	3	22	90	<0.001
	Better	2	2	6	33	
Appearance	Worse	3	1	1	1	
	Stable	9	3	3	135	0.08
	Better	2	2	5	37	
Activity	Worse	2	4	1	10	
	Stable	2	9	8	130	0.009
	Better	-	1	4	31	
Recreation	Worse	1	5	2	10	
	Stable	1	8	4	128	<0.001
	Better	-	-	-	43	
Swallowing	Worse	5	6	6	1	
	Stable	9	16	11	111	<0.001
	Better	1	1	1	34	
Chewing	Worse	5	5	12	9	
	Stable	3	12	23	98	0.009
	Better	2	-	10	23	
Speech	Worse	2	2	4	6	
	Stable	3	4	15	126	0.06
	Better	-	1	6	33	
Shoulder	Worse	4	-	8	4	
	Stable	6	7	12	140	<0.001
	Better	-	-	3	18	
Taste	Worse	5	2	4	2	
	Stable	5	11	10	136	<0.001
	Better	-	2	3	22	
Saliva	Worse	3	11	10	5	
	Stable	8	8	11	126	<0.001
	Better	1	1	1	17	
Mood	Worse	12	8	3	3	
	Stable	4	7	9	126	<0.001
	Better	1	2	3	24	
Anxiety	Worse	12	6	3	6	
	Stable	3	19	12	111	<0.001
	Better	-	1	2	27	

* Significant problem trigger criteria.

Pain, appearance, activity, recreation, mood: (scores of 0 or 25 or 50 & important).

Swallowing, speech, anxiety: (scores of 0 or 30).

Shoulder, taste, saliva: (scores or 0 or 30 & important).

Chewing: (score of 0).

## Results

From 1 August 2007 to 8 July 2009, a total of 202 patients completed the touch-screen package of PCI and UW-QOL at 448 clinic appointments. The mean (SD) age of patients using the touch-screen at the first study clinic was 63 (11) years and these clinics were a median (IQR) of 17 (4–47) months after diagnosis of HN cancer. Of 202 patients 58% (117) were male, 78% (158) had a SCC diagnosis, 72% (138/193) had oral cavity tumours, 78% (143/184) had clinical T1-T2 tumours, 19% (35/186) clinical N positive tumours, 44% (89) had received HN radiotherapy since diagnosis and 48% (96) had had free-flap surgery.

Overall results for patients reporting their condition as worse (W), the same (S) or better (B) over the previous month are shown in Table 1. The only notable differences were observed in relation to time from diagnosis (also Table 1) and there were no significant differences (p < 0.01 chi-squared test) with gender, age at clinic diagnosis (<55, 55–64, 65+) and whether patients ever had radiotherapy or free-flap surgery. The only other significant difference (P = 0.006) was for the saliva domain with tumour site: oral (W 12%, S 80%, B 8%), oropharyngeal (W 23%, S 74%, B 3%), others (W 15%, S 55%, B 30%). For clinics held within 12 months (median 4 months), the trend for all UW-QOL domains apart from saliva and mood (Table 1) was for more improvement than deterioration. In the longer-term (median 50 months) there was an increase in patients reporting stability in their condition for all domains apart from saliva.

For most UW-QOL domains the distribution of patient W-S-B responses differed (p < 0.01) between 4 groups defined by whether patients had significant problems and/or wanted to discuss the issue in the consultation (Table 2). After making allowance for these associations there remained clear residual and notable variation in W-S-B responses. Of patients with a significant pain problem 61% (25/41) said their pain was worse, and hence 39% either stable (29%) or getting better (10%). Similarly for a significant problem with mood, 59% (20/34) said that mood was getting worse and hence 41% either stable (32%) or getting better (9%). For all other domains less than half of patients with a significant problem reported that things were worse (median 33%, range 20-44%, n = 10 domains), thus indicating a minority subgroup for whom a significant problem was worsening. Of those patients without a significant problem but wanting to discuss the issue, there was a minority subset (median 25%, range 8–45%, n = 12 domains) whose condition was getting worse. Finally, and within the vast majority of patients without a significant problem on a particular domain and also not wanting to discuss the issue there was a small subgroup (median 4%, range 0.6–7%, n = 12 domains) of patients whose condition had got worse; for some domains this subgroup claimed around half of all patients whose condition had got worse – activity 10/17, recreation 10/18, speech 6/14.

**Table 2:** Time from diagnosis, and whether for each UW-QOL domain patients had said that things had got worse, stayed the same or got better over the previous month

				**Months from diagnosis to clinic^5^**						**P Value^4^**
		**All patients (202)**		**<12 months^1^**		**12-23 months^2^**		**24+ months^3^**		
		**%**	**N**	**%**	**N**	**%**	**N**	**%**	**N**	
Pain	Worse	17	(35)	21	(18)	17	(4)	13	(11)	
	Stable	61	(124)	56	(47)	57	(13)	72	(61)	0.26
	Better	21	(43)	23	(19)	26	(6)	15	(13)	
Appearance	Worse	3	(6)	4	(3)	4	(1)	1	(1)	
	Stable	74	(150)	67	(56)	74	(17)	85	(72)	0.10
	Better	23	(46)	30	(25)	22	(5)	14	(12)	
Activity	Worse	8	(17)	12	(10)	13	(3)	4	(3)	
	Stable	74	(149)	66	(55)	74	(17)	85	(72)	0.05
	Better	18	(36)	23	(19)	13	(3)	12	(10)	
Recreation	Worse	9	(18)	10	(8)	13	(3)	7	(6)	
	Stable	70	(141)	62	(52)	61	(14)	82	(70)	0.03
	Better	21	(43)	29	(24)	26	(6)	11	(9)	
Swallowing	Worse	9	(18)	8	(7)	4	(1)	11	(9)	
	Stable	73	(147)	71	(60)	70	(16)	79	(67)	0.29
	Better	18	(37)	20	(17)	26	(6)	11	(9)	
Chewing	Worse	15	(31)	18	(15)	17	(4)	13	(11)	
	Stable	67	(136)	58	(49)	70	(16)	79	(67)	0.04
	Better	17	(35)	24	(20)	13	(3)	8	(7)	
Speech	Worse	7	(14)	8	(7)	9	(2)	6	(5)	
	Stable	73	(148)	61	(51)	83	(19)	84	(71)	0.006
	Better	20	(40)	31	(26)	9	(2)	11	(9)	
Shoulder	Worse	8	(16)	11	(10)	4	(1)	6	(5)	
	Stable	82	(165)	74	(62)	83	(19)	89	(76)	0.10
	Better	10	(21)	14	(12)	13	(3)	5	(4)	
Taste	Worse	6	(13)	5	(4)	9	(2)	7	(6)	
	Stable	80	(162)	80	(67)	78	(18)	85	(72)	0.62
	Better	13	(27)	15	(13)	13	(3)	8	(7)	
Saliva	Worse	14	(29)	11	(9)	13	(3)	19	(16)	
	Stable	76	(153)	80	(67)	74	(17)	73	(62)	0.61
	Better	10	(20)	10	(8)	13	(3)	8	(7)	
Mood	Worse	13	(26)	17	(14)	26	(6)	4	(3)	
	Stable	72	(146)	68	(57)	52	(12)	86	(73)	0.003
	Better	15	(30)	16	(13)	22	(5)	11	(9)	
Anxiety	Worse	13	(27)	13	(11)	22	(5)	9	(8)	
	Stable	72	(145)	70	(59)	70	(16)	78	(66)	0.47
	Better	15	(30)	17	(14)	9	(2)	13	(11)	

1: median 4 months, IQR 2-7 months.

2: median 17 months, IQR 15-22 months.

3: median 50 months, IQR 38-82 months.

4: chi-squared test.

5: not known for n = 10.

In all, these 202 patients at 448 attendances selected 1752 items on the PCI that they would like to discuss in consultation, and 58% (1024/1752) of these were issues not covered by the UW-QOL questionnaire. The UW-QOL selections alone through its algorithms and worsening domains could only be matched up with 23% (397/1752) of all PCI items selected by these patients. In addition, UW-QOL algorithms highlighted another 440 significant problems that the patient did not want to discuss (i.e. the corresponding items on the PCI were not selected).

Repeat clinic data were available for 110 patients and 75 of these had repeat clinics that were at most six months apart (median 2.8, IQR 1.8–4.1 months apart, n = 188 pairs of clinics). At the later clinic (clinic 2 in Table 3) most patients (median 73%, range 62–80% over the 12 domains) said their condition had been stable (S) over the previous month, and for these patients their statement was reflected in minimal changes in mean UW-QOL scores between earlier and later clinic pairs (median +1, range −2 to +4, n = 12 domains). Changes in mean UW-QOL scores were higher (median +5, range −2 to +8, n = 12 domains) for patients getting better (B), but were notably lower (median −14, range −30 to −3, n = 12 domains) for those getting worse (W). Also for each domain those getting worse (W) started off at the earlier clinic with scores lower on average than those stable (S) or getting better (B); the tendency was thus for those with the lowest scores at a clinic to get even lower by the next clinic.

**Table 3:** UW-QOL domain scores between 188 pairs of clinic visits (from 75 patients) up to 6 months apart, by whether at the later clinic patients had said that things had got worse, stayed the same or got better over the previous month

	**WBS at clinic 2**	**N**	**UW-QOL mean score at clinic 1**	**UW-QOL mean score at clinic 2**	**Change in UW-QOL domain score from clinic 1 to clinic 2**			
					**Number worse**	**Number same**	**Number better**	Mean change in UW-QOL score*
Pain	Worse	25	56	44	11	12	2	-12
	Stable	116	71	75	16	76	24	+4
	Better	47	84	90	4	28	15	+6
Appearance	Worse	8	59	50	2	6	-	-9
	Stable	136	73	76	18	88	30	+3
	Better	44	77	82	2	32	10	+5
Activity	Worse	15	52	48	3	10	2	-3
	Stable	128	69	66	21	92	15	-2
	Better	45	75	80	5	27	13	+5
Recreation	Worse	14	61	52	5	8	1	-9
	Stable	138	71	72	23	87	28	+1
	Better	36	87	87	9	18	9	0
Swallowing	Worse	13	57	31	8	4	1	-26
	Stable	141	69	71	12	111	18	+2
	Better	34	80	84	3	25	6	+4
Chewing	Worse	22	39	23	7	14	1	-16
	Stable	137	53	55	9	110	18	+3
	Better	29	76	79	2	23	4	+3
Speech	Worse	11	68	59	3	8	-	-9
	Stable	135	79	79	11	112	12	0
	Better	42	75	81	2	31	9	+6
Shoulder	Worse	13	41	34	7	2	4	-7
	Stable	135	78	79	18	96	21	+1
	Better	40	81	85	3	30	7	+4
Taste	Worse	9	43	24	4	4	1	-19
	Stable	150	70	70	20	108	22	0
	Better	29	76	81	1	25	3	+5
Saliva	Worse	21	52	36	8	13	-	-16
	Stable	147	64	63	21	107	19	-1
	Better	20	84	82	3	16	1	-2
Mood	Worse	16	56	27	9	6	1	-30
	Stable	135	73	73	36	66	33	0
	Better	37	82	88	3	25	9	+6
Anxiety	Worse	16	59	36	9	5	2	-23
	Stable	137	68	72	17	86	34	+4
	Better	35	80	88	4	19	12	+8

* Differences between mean scores of clinic 1 and clinic 2 were done at 2 decimal places and then rounded to the nearest unit.

## Discussion

This study introduced the question: ‘*For each domain, please indicate if things had got better, stayed the same or had got worse in the last month*’ into the UW-QOL questionnaire and was used in routine clinical practice at individual patient level. This modification to the UW-QOL had the potential to improve its ability to target key aspects for discussion during the clinic visit. The study results provide evidence in support of this but they also confirm the limitation of relying exclusively on HRQOL tools to identify areas for clinic discussion.

The addition of the W-S-B for each domain may be considered as a natural progression of enhancing the clinical value of using UW-QOL in routine practice. Alternative measures such as the Patient Generated Index (PGI) [26] and the Schedule for the Evaluation of Individualized Quality of Life - Direct Weighting (SEIQoL-DW) [27], are time consuming for routine practice as they involve a 3-stage process; starting with patients nominating domains considered important, then rating their perception of progress in each and then indicating the relative importance of each domain. The overall score obtained is reported as being more representative of the individual’s health status in monitoring response to treatment [28] and in facilitating the discussion of QOL issues [29].

While it is important to obtain an individualized HRQOL measure for monitoring individual response to treatment and aiding patient-clinician communication in routine oncology setting, it is also vital that this measure retains its relevance at group level [30]. When this entire cohort of survivors was evaluated for W-S-B scores, the only consistent differences were in regard to the time since their treatment. Patients within the first year reported more improvement than deterioration in all domains apart from saliva; and as time went on patients demonstrated increasing stability in all domains except saliva. These findings match the experience of HNC patients and their UW-QOL scores in the first 12 months post-treatment [31] and also those of long-term HNC survivors [32]. The results for saliva probably reflect the impact of radiation-induced xerostomia in those receiving radiotherapy [33] and are consistent with the reported deterioration in salivary flow and saliva-associated QOL measures in the first 12 months following treatment, variable recovery in the 12–24 months period and progressive deterioration in the long-term [34,35].

The algorithm trigger criteria used (in Table 2) to identify 'significant' problems on the UW-QOL could have given rise to false negative and false-positive classification. In targeting those without significant problems who are getting worse and those with significant problems who are getting better it may be that the W-S-B serves as a safety net to help pick up false-negatives and well as helping identify less-affected positives.

From Table 2 it can also be observed that in the absence of a significant problem on the UW-QOL, the patient’s perception of things getting worse is more likely to result in that particular domain being selected on the PCI for discussion. The Self-Regulation model of chronic illness [21] might explain this through changes in illness representation, coping strategies, and appraisal of the success of the coping efforts utilising cognitive and emotional regulatory pathways. For example, a patient experiencing pain (i.e. internal cue) who despite best efforts (i.e. coping strategies) has worsened (i.e. appraisal) despite minimal interruption to activities of daily living (i.e. appraisal via cognitive pathway), has experienced anxiety and distress (i.e. appraisal via emotional pathway) and who highlights’pain’ for discussion if it is perceived as a sign that the cancer has returned and is incurable (i.e. illness representation). Clearly, based on this model, HNC patients are more likely to highlight an issue that is perceived to be of personal significance rather than simply the severity of the problem [13,36,37].

Pairs of clinics were included for the change analyses if they were at most six months apart and then for each pair the difference in UW-QOL domain scores were computed. These were compared to W-S-B responses as to whether things had got worse, stayed the same or got better in the last month. Ringash et al. [38] defined a minimal important difference (MID) as the smallest difference that reflects a clinically important change in score and noted that most published estimates of MID fall into the range 5–10% of the instrument range. In our study, changes in mean UW-QOL scores for those getting worse (W) were typically lower by 10 or more units within a scale of 0 to 100. This would imply at group level clinically important changes between clinics. The narrow range of changes in score associated with patients reporting stability fall below Ringash's 5–10% threshold for defining a minimally important clinical difference. The W-S-B has the advantage of providing this information from a single visit rather than from two without the need for additional computations. This analysis was limited by repeat review clinics being almost always more than one month apart, with data that were not independent because some patients contributed more pairs of clinics than others. It was also conceivable that a patient could indicate a change had occurred on the W-S-B and for that change not to show in the UW-QOL domain scores, this reflecting a lack of precision in the UW-QOL single question domains and their small number of levels of discrimination.

We found that only 42% of items highlighted on the PCI overlapped with UW-QOL domains and only 23% were through UW-QOL algorithms or worsening domains. Thus there is clear value to using the PCI in addition to the UW-QOL in identifying a wider range of key concerns for discussion. These issues include fear of recurrence in particular, and others like sleeping, energy levels, lifestyle issues, support for family and financial concerns [23].

If both UW-QOL and PCI are used together then the W-S-B question can identify significant problems that have got worse, and non-significant problems that patients want to discuss and that have got worse. For professionals not intending to use the PCI and only the UW-QOL then the W-S-B targets significant problems that have got worse and non-significant problems that have got worse. Depending on actual domain scores it might be that appropriate intervention may prevent a worsening situation becoming a significant problem.

The study is limited in that the study population was from a single consultant whose practice may not be typical of others. Also we only analysed items as identified by the touch-screen before consultation and did not consider items actually discussed between the patient and clinician. We also did not seek patient feedback about the W-S-B question.

In conclusion, this study found that the W-S-B question adds little questionnaire burden and could help to better identify patients who might benefit from intervention. The UW-QOL questionnaire through its use of UW-QOL algorithms and worsening domains through W-S-B could only identify about one-quarter of all items selected by patients on the PCI for discussion, which suggests that the UW-QOL and PCI should be used together to allow optimal identification of issues for patient-clinician discussion during routine outpatient clinics.

## Competing interests

The authors declare that they have no competing interests.

## Authors’ contribution

NG was involved in data analysis and interpretation; manuscript preparation, editing and review. DL was involved in developing study concepts and design; data analysis and interpretation; quality control of data and algorithms; statistical analysis; manuscript preparation, editing and review. SNR was involved in developing study concepts and design; data collection, analysis and interpretation; quality control of data and algorithms; manuscript preparation, editing and review. All authors have read and approved the final manuscript.
